# 用于食品安全分析样品前处理的共价有机聚合物的制备及应用进展

**DOI:** 10.3724/SP.J.1123.2020.08013

**Published:** 2021-02-08

**Authors:** Pingli WANG, Yanlong CHEN, Yuling HU, Gongke LI

**Affiliations:** 1.中山大学化学学院, 广东 广州 510275; 1. School of Chemistry, Sun Yat-sen University, Guangzhou 510275, China; 2.广东石油化工学院化学学院, 广东 茂名 525000; 2. School of Chemistry, Guangdong University of Petrochemical Technology, Maoming 525000, China

**Keywords:** 共价有机聚合物, 食品安全分析, 样品前处理, 综述, covalent organic polymers, food safety analysis, sample pretreatment, review

## Abstract

食品安全关系身体健康和生命安全,是全球关注的热点之一。食品基质复杂,痕量有毒有害物质分析之前必须经过有效的前处理。目前发展的前处理技术如固相萃取、磁固相萃取、固相微萃取等,其关键是吸附介质。共价有机聚合物是一类通过共价键连接而成的有机多孔材料,具有质轻、稳定性好、比表面积大、结构可控、易于修饰等特性,是一类优异的新型吸附材料。该文综述了近年来共价有机聚合物(COPs)在食品安全分析前处理中的应用进展。共价有机聚合物及其功能化复合材料通过简单的装填、聚合反应或化学键合固定到小柱或毛细管柱中用作固相萃取的吸附介质;通过一锅法、原位还原法、原位生长法或共沉淀法生成具有磁性的固相萃取吸附介质;或者通过物理涂覆、化学键合、溶胶凝胶法及原位生长法制备固相微萃取纤维。基于以上高吸附容量共价有机聚合物的样品前处理技术,食品中农残兽残、添加剂、环境污染物及生物毒素等得到了有效富集。最后,展望了COPs在食品分析样品前处理应用中的发展方向:简单高效绿色制备方法的开发,功能化COPs的设计合成;萃取机理的研究;高通量、高灵敏度分析方法研究。这些研究将促进COPs在样品前处理领域获得更广泛的应用。

## Abstract (en)

Food safety is closely related to human health and life. Contaminated foods may result in illness or poisoning. For example, perfluorinated compounds can concentrate in the human body, or they can be transferred to the baby during breastfeeding, thus leading to serious health risks. Phthalate esters may cause damage to the liver, lungs, and kidneys. Therefore, food safety has become a hot topic at a global level. Poisonous and harmful substances in foods are derived from the environment, planting or breeding, food contacting materials, and food processing, or due to unsuitable storage conditions. Residues of pesticides and veterinary drugs, organic pollutants, additives, heavy metals, and biotoxins often hamper food safety, causing diseases or even death. The diversity of available food species, complexity of the sample matrix, and lack of information about the source of pollutants render the direct determination of food contaminants difficult. Pretreatment is vital for the accurate analysis of trace toxins in foods. Optimal pretreatment can not only improve the extract efficiency and determination sensitivity, but also prevent instrument contamination. Pretreatment techniques have played an important role in trace determination for complex matrices. Pretreatment methods can be classified as solvent-based and adsorption-based methods. Adsorption-based techniques such as solid-phase extraction, magnetic solid-phase extraction, and solid-phase microextraction are simple and efficient, and hence, are widely used. In these pretreatment techniques, adsorbents play a key role in the extraction effect. In the last few years, metal organic frameworks, metal oxide materials, carbon nanotubes, graphene, and magnetic nanoparticles, as well as a combination of these materials, have been used as adsorbents. These materials are porous and have a large surface area; they are used to enrich trace targets and eliminate interferents. Covalent organic polymers (COPs) are a class of organic porous materials constructed from organic monomers via covalent bonding. Given their excellent characteristics such as light density, good stability, high surface area, structural controllability, and ease of modification, COPs are potential adsorbents. COPs are often synthesized by solvent thermal methods. However, these methods are time-consuming and require toxic solvents and harsh reaction conditions. As alternatives, room-temperature methods, mechanical chemical methods, microwave-assisted methods, and UV-assisted methods have been developed. This has facilitated the synthesis of a wide range of COPs. In this article, the recent applications of COPs in sample pretreatment for food safety analysis are reviewed. COPs can be used in solid-phase extraction by simple packing into columns, polymerization, or chemical bonding in the capillary. Magnetic compounds have been prepared by one-pot synthesis, *in situ* growth, *in situ* reduction, or coprecipitation methods and used in magnetic solid-phase extraction. Coatings of solid-phase microextraction fibers are fabricated by physical methods, chemical bonding, sol-gel methods, or *in situ* growth. Toxic and harmful substances in foods and foodstuffs are efficiently extracted by exploiting the high adsorbent capacities and specificity of COPs. Future development prospects and challenges in sample pretreatment are also discussed herein. There is increased focus on the development of simple, efficient, and environment-friendly methods to synthesize COPs with specific functions; further, high-throughput, sensitive analytical methods may be established. In the future, more specific COPs will be prepared in a cost-effective manner for widespread use in sample pretreatment.

共价有机聚合物(covalent organic polymers, COPs)是一类新型的多孔有机材料,完全由碳氢氧氮硼等轻质元素通过共价键连接而成。经过近十多年的发展,科学家们已经设计并制备出了各种各样的共价有机多孔聚合物。目前发展的共价有机多孔聚合物大致分为两大类:一类是晶型的共价有机多孔聚合物,称为共价有机框架材料(covalent organic frameworks, COFs);另一类是无定型的共价有机多孔聚合物。和传统的无机多孔材料如硅藻土、沸石、活性炭等相比,共价有机多孔聚合物具有以下优点:以有机小分子为单体,结构可控和功能可调;通过共价键连接而成,热稳定性和化学稳定性好;全部由轻质元素组成,密度低;比表面积高,吸附容量高。由于具有这些独特优势,COPs在催化^[1]^、传感、环境污染物的吸附去除^[2,3]^、分离^[4]^等领域获得了广泛应用。

食品安全关系人的身体健康和生命安全,是当今研究热点之一。食品安全研究中主要关注的有毒有害物质来源有:食材在种植养殖过程中,不合理使用农药兽药,或者环境中污染物的迁移转化;食品在生产过程中,非法添加或过量添加的化合物;以及由于储存不当或储存时间过长,产生的霉菌毒素;或者食材自身含有的生物毒素等。食品基质复杂,而且有毒有害物质以痕量存在,大大增加了食品安全分析的难度。近年来发展的样品前处理技术如固相萃取、磁固相萃取、固相微萃取等可以去除基质干扰、富集痕量目标物,提高痕量分析的准确度和灵敏度。吸附介质是这些样品前处理技术的关键,具有高吸附容量的COPs是潜在的优异吸附介质。Yang等^[5]^就共价有机骨架材料作为气相色谱、高效液相色谱和毛细管电色谱的固定相,及其在食物、环境、生物等样品前处理中的应用进行了综述。最近,Bai等^[6]^也对共价有机骨架材料在食物、环境、生物等样品前处理中的应用进行了综述。这充分展示了共价有机框架材料在分析化学领域的巨大潜力。结合本课题组的研究工作,本文主要综述COPs在食品安全分析样品前处理中的应用。

## 1 COPs在固相萃取中的应用

固相萃取(solid-phase extraction, SPE)通常是将吸附剂装填到柱内,用合适溶剂进行活化、上样、淋洗、洗脱等过程的前处理技术。将COPs以粉末形态装填到小柱中,或者将合成COPs的反应溶液灌装到预先处理好的毛细管中制备成含有COPs的整体材料等方式,制得基于COPs的SPE柱。

本课题组Zhang等^[7]^基于席夫碱反应,以对苯二甲醛与1,3,5-均苯碳酰肼为原料通过缩聚反应制备了腙键连接的共价有机聚合物。该材料具有疏水性,苯环和亚胺结构使其含有丰富的*π-π*堆积电子,可形成氢键,可与缺电子化合物发生电子给予-接收相互作用。因此,该材料对目标物具有很强的亲和识别作用。与商品化吸附剂相比,它的吸附容量是商品化吸附剂(C_18_硅胶吸附剂、多壁碳纳米管、石墨烯)的1~11倍。将其用作SPE吸附介质制作SPE微柱,SPE微柱连入进样管路中,通过切换进样阀实现与HPLC在线联用。他们利用构建的在线SPE-HPLC系统对干辣椒粉和香肠中6种苏丹红染料进行分析,检出限低至0.03~0.15 μg/L,富集因子可达305~757。先制备材料,再将材料粉末装入小柱中制作SPE柱的方式存在吸附剂流失和制备麻烦的缺点。因此,本课题组对此进行改进,制备了含有COPs的整体材料为吸附介质的SPE^[8]^。以均苯三甲酰肼和对苯二甲醛为原料,通过原位化学键合策略在石英毛细管中制备了酰腙键连接的动态共价聚合物凝胶整体材料,并利用该材料构建了在线SPE-HPLC分析系统,用于在线富集和分离。将其用于食品(鲈鱼、虾、袋装牛奶、茶叶)中4种荧光增白剂和6种磺胺类抗生素的富集分析,荧光增白剂的富集因子为50~66,检出限为0.3~1.0 μg/L;磺胺类抗生素的富集因子为72~124,检出限为0.05~0.30 μg/L。该材料对含有*π-π*结构和疏水特性的目标物具有很好的亲和吸附性能,是一种有潜力的样品前处理介质。

由于共价键的稳定性,酰腙键连接的共价交联聚合物凝胶比超分子网络更稳定,具有良好的通透性和重现性。以三[(4-甲酰苯氧基)甲基]乙烷和对苯二甲酰肼为单体,本课题组Wei等^[9]^制备了共价交联聚合物凝胶整体柱,并首次将共价交联聚合物凝胶用于食品中黄曲霉素的在线富集。该材料通过疏水作用、*π-π*堆积以及立体效应对黄曲霉素具有很好的吸附富集作用,基于此建立的分析方法检出限是0.08~0.2 μg/kg,相对标准偏差(RSD)是1.1%~9.6%,回收率为76.1%~113%;在阳性豆酱中检测到黄曲霉素G1和B1分别是32.8 μg/kg和26.4 μg/kg;大豆中黄曲霉素G1含量是25.9 μg/kg。基于共价交联聚合物凝胶良好的萃取效果,又成功用于柑橘类水果中氯吡磷和三唑磷在线萃取富集与分析^[10]^。此外,研究者还以三苯基化合物为反应单体、选择合适的交联剂制备共轭微孔聚合物^[11,12]^,将其用于西红柿和黄瓜中苯甲酰脲类农药的富集处理,回收率为80.8%~118%,检出限为0.03~0.05 ng/g,线性范围为0.5~100.0 ng/g。随后,将该材料用于瓶装饮料和西红柿中氯酚农药的吸附富集,检出限为0.03~0.3 ng/mL,饮料中氯酚的富集因子为127~183,西红柿中其富集因子为110~150; RSD小于5.7%;添加回收率为92.5%~106.3%。上述研究表明,以各类含苯环结构的化合物为单体,通过席夫碱反应制备多孔有机聚合物,将其作为食品前处理中的吸附介质,并与色谱联用,可以准确灵敏地分析食品中痕量有毒有害物质。

利用席夫碱反应制备共价有机聚合物的制备过程繁琐耗时,王志等^[13]^开发了机械研磨法快速制备COFs。他们以1,3,5-三甲酰间苯三酚和对二氨基偶氮苯为单体,与对-甲苯磺酸一起研磨,然后置于170 ℃加热60 s,冷至室温后用去离子水洗去过量的对甲苯磺酸,然后用*N*,*N*-二甲基乙酰胺、丙酮、水依次清洗,烘干制得共价有机框架材料1,3,5-三甲酰间苯三酚-对二氨基偶氮苯。用该材料作为吸附剂,富集果汁和西红柿中的苯甲酰脲类农药,回收率达84.1%~108.6%。而且,果汁中苯甲酰脲类农药的检出限为0.1~0.2 ng/mL,线性范围是1.0~160.0 ng/mL;西红柿中苯甲酰脲类农药的检出限为0.05~0.1 ng/g,线性范围是0.5~80.0 ng/g。随后,该课题组^[14]^以2,6-二氨基蒽醌和1,3,5-三甲酰间苯三酚为单体,通过机械研磨法制备了共价有机框架材料1,3,5-三甲酰间苯三酚-2,6-二氨基蒽醌。将该材料用于果汁、蔬菜水果中苯甲酰脲类农药的富集,后经HPLC分析。果汁中苯甲酰脲类农药的检出限为0.02~0.05 ng/mL,蔬菜水果中为0.02~0.08 ng/mL;线性范围是0.1~160 ng/mL, RSD为2.7%~6.8%。为了提高材料的特异性识别能力,Ji等^[15]^将制备COFs的单体和模板分子混合,室温下以三氟甲基磺酸钪为催化剂,合成了分子印迹聚合物修饰的COFs。用于蔬菜、水果以及中药中氰基拟除虫菊酯农药的选择性富集,富集后经HPLC分析,检出限为0.011~0.018 ng/g,线性范围为0.1~200 ng/g,添加回收率为94.3%~102.7%。

为了比较COFs与无定型COPs的萃取效果,Wang等^[16]^以三苯基苯作为单体,二氯甲烷作为交联剂,同时制备了晶型的层状多孔有机框架和无定型多孔COPs,用于蜂蜜中氯酚的萃取。研究结果表明,由于层状多孔有机框架具有更大的比表面积和微孔体积,因此具有更高的吸附容量,其萃取效果优于无定型多孔共价有机聚合物。与商品化吸附剂(聚苯乙烯、C_18_硅胶材料、多壁碳纳米管)比较,层状多孔有机框架和聚苯乙烯的萃取效果相当,比C_18_硅胶材料和多壁碳纳米管具有更好的萃取效果。

无机离子也是食品安全的研究对象之一,普通的吸附介质萃取效果有限。Liu等^[17]^制备了羧基修饰的1,3,5-三甲醛间苯三甲酸,以它和4,4'-联苯胺为单体通过加热回流制备了新型COFs。将羧基化COFs粉末装入小柱中,两端用玻璃棉封堵,作为萃取无机离子的SPE微柱,构建了在线富集分析系统微固相萃取-电感耦合等离子体-质谱法(μSPE-ICP-MS),通过流动注射方式,实现了水、牛奶中铬(Ⅲ)、锰(Ⅱ)、钴(Ⅱ)、镍(Ⅱ)、镉(Ⅱ)、钒(Ⅴ)、铜(Ⅱ)、砷(III)、硒(Ⅳ)和钼(Ⅵ)等10种离子的在线富集分离检测。该方法的检出限低至2.1~21.6 ng/L,线性范围是0.05~25 μg/L, RSD是1.2%~4.3%。

## 2 COPs在磁固相萃取中的应用

磁性物质作为吸附剂,分散到样品溶液中,经过超声或振荡混匀并孵化一定时间,目标物被吸附。然后在外加磁场作用下,吸附剂快速与基质溶液分离。吸附有目标物的吸附剂经过洗脱,将目标物与磁性吸附剂分离。这样的前处理技术称为磁性固相萃取(magnetic solid-phase extraction, MSPE)。将磁性物质与比表面积大、孔洞丰富、吸附容量高的COPs结合形成磁性COPs复合材料,具备磁性材料的方便操作和COPs的高吸附特性,可以快速高效地吸附富集目标物并通过外加磁场与基质分离。

本课题组Lei等^[18]^将氨基化四氧化三铁磁性纳米颗粒通过硅烷偶联剂连接上亚苯基乙炔基,然后再通过Sonogashira反应与三溴苯和三乙炔苯交叉偶联,获得由亚苯基和亚乙炔基组成的具有三维网络结构的磁性聚亚苯基亚乙炔基的共轭微孔聚合物材料。该材料具有强共轭体系、超顺磁性,可以富集含有共轭结构的痕量化合物并实现方便快捷的磁分离。基于材料的富集特性和磁性,用于水果蔬菜中6种杀菌剂的萃取,建立了UPLC-MS/MS分析方法。该方法检出限为0.27~3.1 ng/L, RSD为1.1%~3.9%。结果表明,磁性聚亚苯基亚乙炔基的共轭微孔聚合物可以方便地富集水果蔬菜中痕量的含多个苯环的农药。此外,利用材料的*π-π*堆积和疏水特性,还将其用于富集果蔬中具有芳香族共轭结构的有机磷杀虫剂^[19]^,建立了果蔬中灵敏准确的有机磷杀虫剂分析方法。此外,Liang等^[20]^还以2,4,6-三(4-氨基苯基)-1,3,5-三嗪和对苯二甲醛为原料,制备了三嗪-亚胺杂交的核壳型磁性COPs,该材料具有*π-π*共轭结构和丰富的孔径,将其用于水果中含共轭结构农药的萃取,建立了水果中8种农药的MSPE-UPLC-MS/MS分析方法,检出限为0.4~1.2 ng/L,同一批材料的RSD为0.7%~7.0%,不同批次材料间的RSD为1.7%~10%。

本课题组Liang等^[21]^通过光化学反应快速制备了磁性碳纳米管与COFs的复合物。首先在碳纳米管内沉积磁性纳米颗粒形成磁性碳纳米管,碳纳米管经纯化处理生成的羟基可以和对苯二硼酸反应,紫外光照条件下在磁性碳纳米管表面发生界面合成形成磁性COFs与碳纳米管的复合物。磁性颗粒沉积在碳纳米管内,有利于其外部活性位点充分暴露,增加COFs的键合量。在碳纳米管表面进行COFs界面合成,可以增加碳纳米管的分散性,避免碳纳米管团聚,有效提高材料的表面积。光化学合成制备COFs的显著优势是反应条件温和,反应时间由通常的72 h缩短到48 h。该复合材料具有高特异性比表面积,碗形的超分子识别结构以及缺电子的硼氧键,这些特征使其可以高效富集杂环胺。将其用于炸鸡、烤牛肉中杂环胺的吸附富集,烤牛肉中杂环胺的添加回收率为73.0%~117%, RSD为1.5%~8.9%;炸鸡中杂环胺的添加回收率为76.3%~108%, RSD为1.3%~9.1%。结果表明,该材料对于实际样品中的杂环胺具有高特异性吸附。

Li等^[22]^先用单体联苯胺修饰磁性四氧化三铁纳米颗粒,然后匀速滴加1,3,5-三甲酰间苯三酚的四氢呋喃溶液,通过加热回流制备核壳型磁性共价有机框架材料。材料的制备过程及在MSPE的应用示意图见图1。该材料磁饱和度高,对外加磁场反应迅速。增强的比表面积和孔隙特征,利于其高容量吸附目标物,已成功用于食品中痕量多环芳烃的富集。

**图1:**
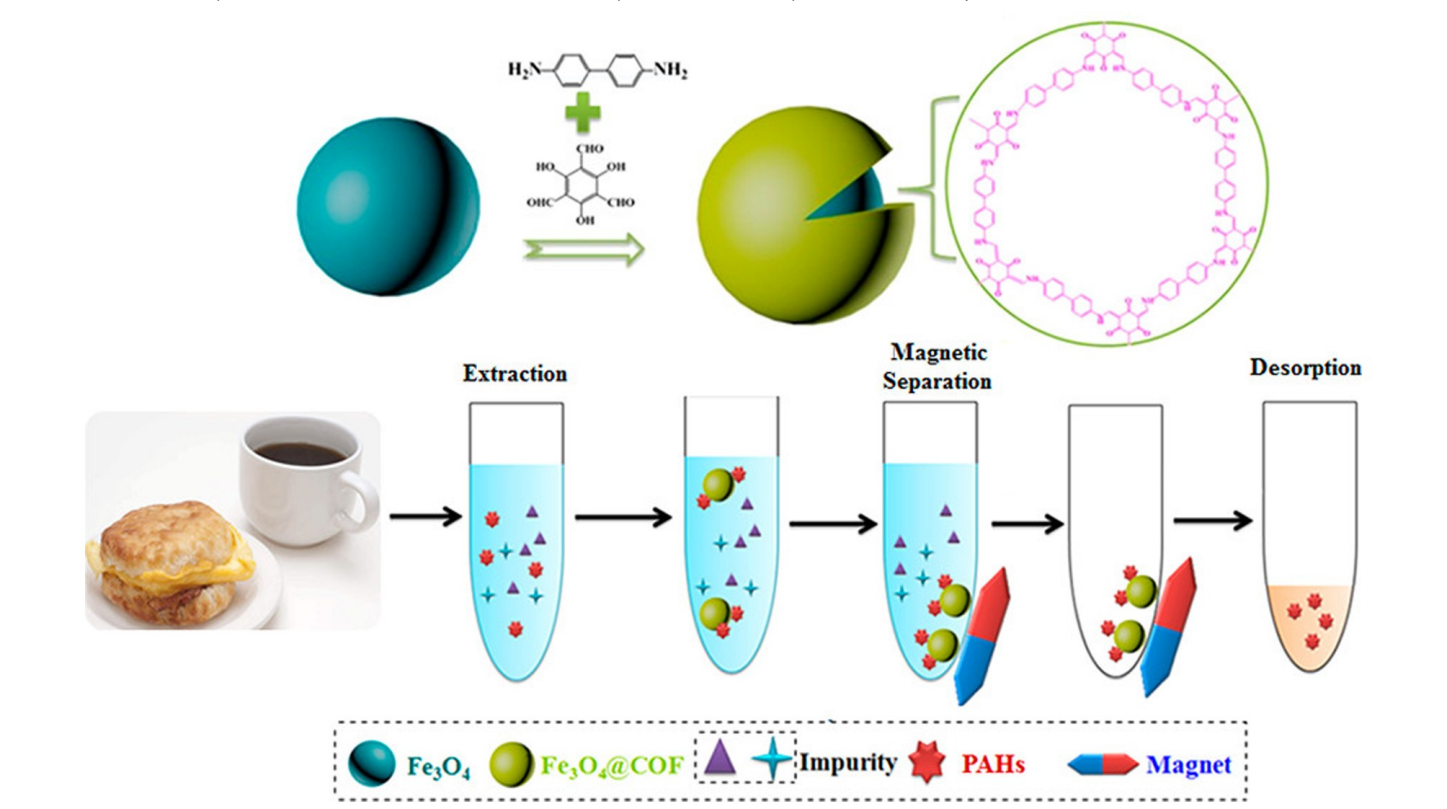
磁性共价有机框架复合材料制备过程及其MSPE应用示意图^[22]^

Zhao等^[23]^采用后修饰策略制备了磺酸基功能化的疏水亲脂/阳离子交换吸附磁性共价三嗪框架材料。具体制备过程如下,以具有大比表面积、易于后修饰的框架结构材料共价三嗪框架材料作为平台,嫁接磺酸基;然后将负载在共价三嗪框架材料上的镍离子通过原位还原的方法赋予材料强磁性特征。以磺酸基修饰的磁性共价三嗪材料为吸附介质,对水果蔬菜以及果汁中苯并咪唑类农药进行多模式吸附的富集萃取,经HPLC分析,检出限为1.23~7.05 μg/kg,添加回收率为80.2%~115.1%, RSD为4.9%~11.5%。

与普通COFs对目标物的吸附作用相比,功能化COFs与目标物具有更强的亲和作用,因此具有更好的选择性吸附能力。最近,Zhang等^[24]^以1,3,5-三甲醛间苯三酚(TP)和2,3,5,6-四氟-对苯胺为单体,通过溶剂热法制备了氟功能化的磁性COFs。该材料具有规则的孔径、高比表面积和较强的磁性,用于牛奶中6种全氟化物的MSPE-LC-MS/MS分析检测,富集因子可达21.91~100.6,检出限为0.005~0.05 ng/L,添加回收率为81.31%~128.07%,日间RSD为0.5%~11.8%,批间RSD为2.3%~11.7%。为了比较材料的特异性吸附能力,他们同时研究了纳米磁性四氧化三铁和普通磁性核壳型COFs对全氟化物的吸附效果。研究结果表明,经普通磁性核壳型COFs吸附后的样品中全氟化物含量低于磁性四氧化三铁吸附后的样品,但经氟功能化的磁性COFs吸附后的样品中全氟化物基本无检出。这充分说明氟功能化的磁性COFs对全氟化物具有优秀的特异性吸附能力。

## 3 COPs在固相微萃取中的应用

固相微萃取(solid-phase micro extraction, SPME)是一种将取样、富集、进样为一体的样品前处理技术。通过物理涂覆、溶胶-凝胶或化学键合的策略将吸附介质固载到石英纤维或不锈钢钢丝上,或者在纤维上原位生长吸附介质。SPME萃取高效、不需额外溶剂,样品用量少,在复杂基质样品预处理中应用广泛。

本课题组Pan等^[25]^将三聚氰胺和对苯二甲醛分散到二甲基亚砜中,在程序温控下进行微波反应得到白色的席夫碱网络结构聚合物。通过硅烷偶联剂,采用共价桥连策略将席夫碱网络结构聚合物键合到石英纤维上,形成牢固稳定的富集涂层,制备过程见示意图2。将基于席夫碱网络结构聚合物作吸附层的SPME纤维依次在乙醇、己烷、沸水、氢氧化钠溶液、硫酸溶液中浸泡5 h,对富集效果没有明显影响,表明通过共价键高度交联的席夫碱网络结构聚合物具有优异的热稳定性和化学稳定性。席夫碱网络结构聚合物对具有共轭结构的化合物有强亲和作用,可富集多环芳烃;材料富含氨基,可利用酸碱作用富集含有羧基的挥发性脂肪酸。将其用于茶叶和烟叶中挥发性脂肪酸富集,并进行GC-MS分析,方法检出限为0.014~0.026 μg/L, RSD为4.3%~9.0%。与商品化SPME纤维相比,以席夫碱网络结构聚合物为吸附层的SPME纤维具有更高的富集因子。这充分说明该材料具有强特异性吸附能力。

**图2:**
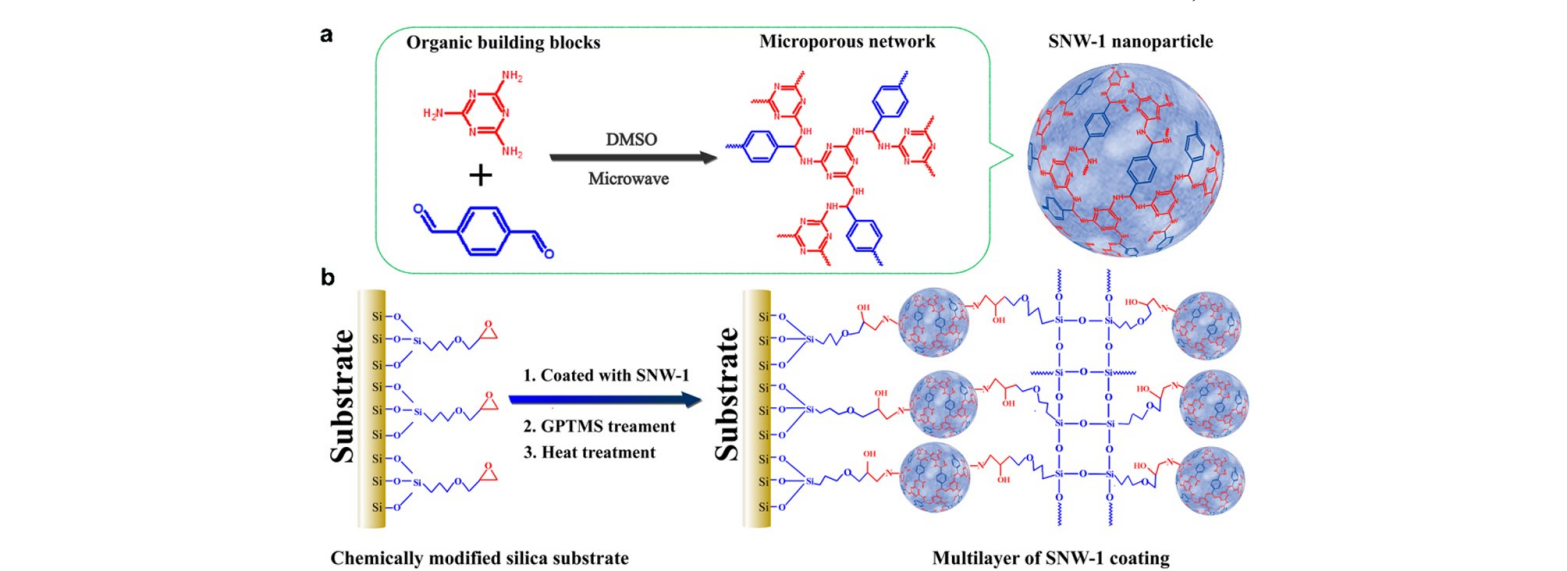
(a)SNW-1及(b)富集涂层的制备示意图^[25]^

最近,Peng等^[26]^以四(对溴苯基)卟啉和对苯基二硼酸为原料,用微波辅助法快速合成了卟啉共轭微孔聚合物;以卟啉共轭微孔聚合物为吸附介质通过化学键合法制备SPME萃取纤维。基于吸附介质合适的微孔结构、高比表面积以及与极性化合物之间的氢键相互作用,该SPME对烟叶中挥发性酸具有优异的富集效果,富集因子高达66657~133970,检出限低至0.0046~0.022 μg/L,成功用于烟叶中痕量挥发性酸的富集分析。

王志等^[27]^用盐酸溶液刻蚀不锈钢钢丝增加表面积,通过溶胶-凝胶法将利用机械研磨制备的共价有机框架材料(COF-TPBD)涂覆到不锈钢钢丝上制得SPME吸附层;该SPME纤维成功用于蜂蜜、黄桃罐头等样品中氯酚萃取;与商品化PA、PDMS、PDMS/DVB萃取纤维相比,COF-TPBD纤维的富集倍数约为商品化的2~7倍,具有更高的萃取效率。此外,他们^[28]^用同样的方法制备了COF-(TpPa-NO_2_)萃取纤维,高效富集了蔬果中残留的农药,检出限为0.04~0.25 μg/kg, RSD小于11.2%时添加回收率为81.5%~111%。Wu等^[29]^采用多巴胺修饰不锈钢钢丝,通过多巴胺将腙型COFs固载到不锈钢钢丝上制备SPME纤维,用于蔬果中拟除虫菊酯的预富集,富集因子高达307~2327,检出限为0.11~0.23 μg/kg,批内RSD为3.6%~9.2%,批间RSD为6.9%~12.1%。该课题组^[30]^还通过光诱导硫醇-烯键点击化学制备交联COFs用作SPME吸附层,用于有机氯农药的预处理,富集因子高达2190~10998,检出限为0.0003~0.0023 ng/kg,批内RSD为3.4%~7.6%,批间RSD为5.7%~11.6%,添加回收率为78.2%~107%。此外,他们^[31]^还用共价三嗪框架材料作为吸附剂,用物理涂覆的方法制备SPME,用于邻苯二甲酸酯类化合物的萃取,富集因子高达7790,检出限为5~95 ng/L,批间RSD为3.1%~10.9%,批内RSD为0.8%~4.7%。

物理涂覆制作SPME吸附层,操作简便,但涂层不稳定。Guo等^[32]^开发了化学键合、原位生长COFs吸附层制备方法。他们用3-氨丙基三乙氧基硅烷修饰不锈钢钢丝,然后将单体TP与氨基键合,最后在室温环境下生成COF-TPBD吸附层,COFs合成及SPME制备见图3。与商品化SPME涂层PDMS(30 μm)、PDMS/DVB(65 μm)相比,COF-TPBD(10 μm)对多氯联苯具有更高的富集因子。这主要是基于疏水作用、*π-π*堆积、立体空间位阻效应等共同作用的结果。基于此,他们成功建立了多种水产品中多氯联苯的HS-SPME-GC-MS/MS分析方法。与其他分析方法相比,该方法具有更低的检出限,低至0.07~0.35 ng/L,富集因子为4471~7488,加标回收率87.1%~99.7%。上述研究表明,以COFs为吸附层的SPME纤维,具有良好的热稳定性和化学稳定性,可用于复杂基质中痕量目标物的高效萃取富集,并建立准确灵敏的检测方法。

**图3:**
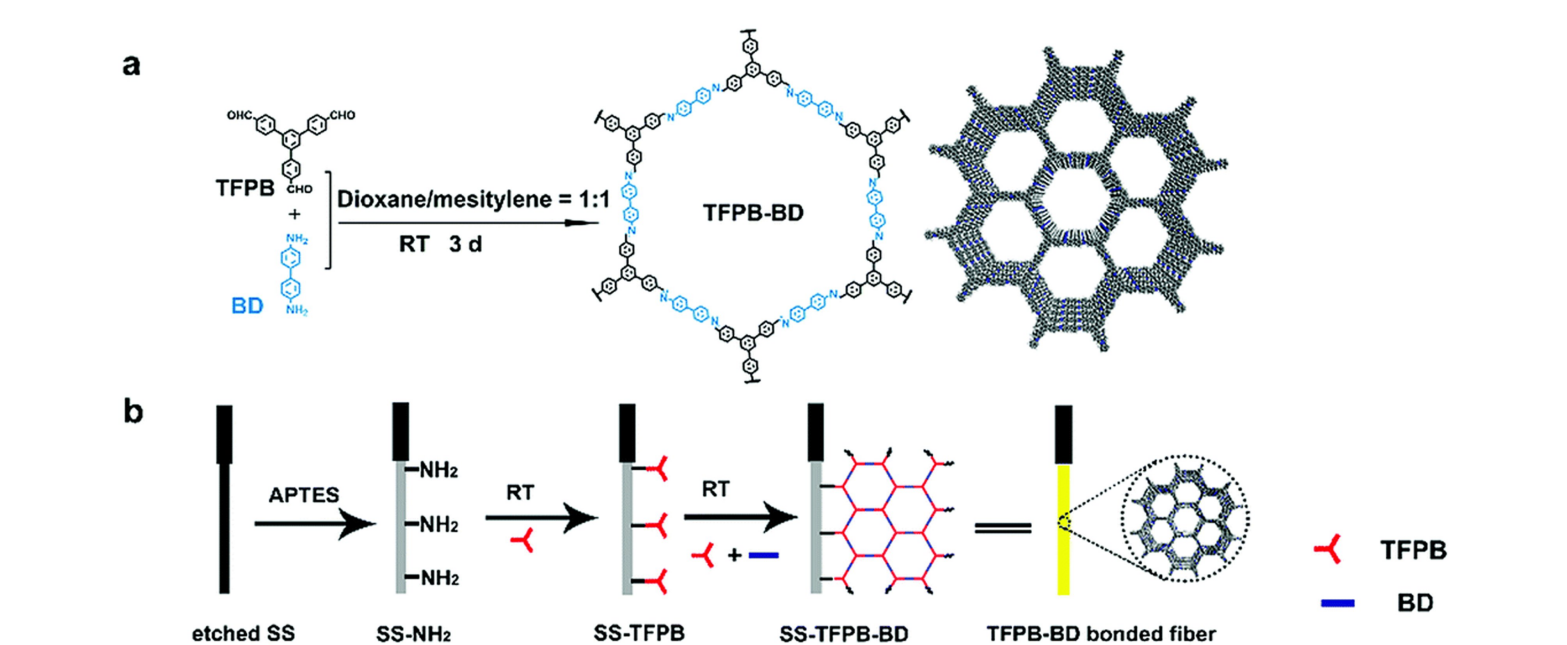
(a)经TFPB和BD缩聚室温合成TFPB-BD与(b)室温制备TFPB-BD化学键合SPME纤维的示意图^[32]^

## 4 COPs在其他前处理中的应用

在传统柱式SPE基础上,研究者开发了更方便、成本更为低廉的移液吸管头式SPE(pipette tip SPE, PT-SPE)。以PT作为SPE的柱体,将吸附剂装填在PT中制成PT-SPE,可以节省吸附剂用量,满足痕量样品制备目的。Chen等^[33]^以金属有机框架-共价有机框架杂交材料作为吸附剂制备了PT-SPE,用于萃取水、牛奶、肉等食品中的磺胺类药物。单纯以COFs作为吸附剂制备PT-SPE,吸附剂易流失,纳米级颗粒易引起堵塞,不适合大量样品快速上样。Yan等^[34]^以三聚氰胺和对苯二甲醛为单体制备了席夫碱网络结构框架材料(SNW-1),然后利用静电纺丝技术制备聚丙烯腈-席夫碱网络结构框架材料的复合物(polyacrylonitrile@SNW-1, PAN@SNW-1),并以此制作了PT-SPE。基于SNW-1与磺胺类药物之间的强*π-π*相互作用,成功用于鸡肉和猪肉中磺胺类药物的吸附富集。随后,Wang等^[35]^也制备了以PAN@COF-SCU1为吸附剂的PT-SPE,用于草蟹和鸭子中四环素的萃取。基于四环素类药物与COF-SCU1之间的静电作用、*π-π*相互作用、氢键、疏水作用,建立了以PAN@COF-SCU1为吸附剂的PT-SPE-LC分析方法,线性范围是4~70 ng/mL,检出限为0.6~3 ng/mL,定量限为2~10 ng/mL,日内日间RSD均小于9%。这表明,以PAN@COF-SCU1为吸附剂的PT-SPE可以高效富集草蟹和鸭肉中的四环素类药物,基于此建立的分析方法具有较高的回收率和准确性。

痕量目标物经过样品前处理后,与光传感检测结合,可获得快速简便灵敏的分析方法。Zhang等^[36]^利用一锅法反相微乳化聚合制备了量子点和分子印迹双功能化的COFs复合材料,用于富集发酵肉制品中的酪胺。经过富集后,同时用荧光传感器和HPLC检测酪胺。荧光传感检测的线性范围是35~35000 μg/kg,检出限为7.0 μg/kg;经HPLC分析的线性范围是20~2000 μg/kg,检出限为5 μg/kg。这表明,利用这种复合材料处理样品后直接进行荧光传感检测是一种快速、选择性良好的检测方法。Zhang等^[37]^也制备了相似的复合材料,以Tp和Pa为单体通过溶剂热法制备COF-TpPa,然后以杀虫双为模板分子,通过一锅法在室温下合成。将其用于自来水中沙蚕毒素类杀虫剂的吸附富集并进行荧光光谱分析,最大吸附量为771 mg/g,检出限低至1.6 μg/L,线性范围5~100 μg/L和200~2500 mg/L,添加回收率为86.5%~106.5%。量子点和分子印迹共同修饰的COFs复合材料在选择性富集目标物后进行荧光光谱分析,不仅检出限低,分析方法灵敏,且可避免紫外光谱分析中生物基质的干扰,为复杂食品基质中类似物的分析检测奠定了基础。

共价有机聚合物具有高比表面积、易于功能化修饰等特征,将其装填到小柱中,或者赋予其磁性特征,或者制成SPME涂层等,作为样品前处理技术中的吸附介质富集复杂基质中的痕量目标物具有高效、特异性强等优势。这类材料在食品安全分析中样品前处理方面的详细应用见表1。

**表1:** COPs在食品安全分析样品前处理中的应用

Analytes	Samples	Pretreatment	Adsorbents	Analytical methods	Reference
Carbamate pesticides	milk, white wine, juice, lemonade	SPE, MSPE	COPs-BD-THB, M-PCTP	HPLC-DAD or MS	[38,39]
Neonicotinoids	cucumber, lettuce	SPE	Fe_3_O_4_@TPBD-(NO_2_)_2_	HPLC	[40]
Pyrethroids	vegetables, fruits, traditional Chinese medicines	SPE	1,3,5-benzenetricarboxaldehyde-tereph- thalicdihydrazide, MICOF(TPBA-TP)	GC-ECD, HPLC	[15,29]
Benzimidazoles	vegetable, fruit, juice	MSPE	Ni@CTF-SO_3_H, Fe_3_O_4_@TPBD	HPLC	[23,41]
5-NDZs	drinking water	MSPE	CC-TPB	UPLC-MS/MS	[42]
OPPs	grapes	MSPE	Fe_3_O_4_@DtTb	LC-MS/MS	[43]
OCPs	vegetables, fruits	SPME	TpPa-NO_2_, CC-TPA, 1,3,5-benzenetricar- bohydrazide/4-hydroxyisophthalaldehyde	GC-ECD	[28,30,44]
CPs, phenols	honey, beverages, canned-yellow-peach	SPE, MSPE, SPME	CMPs-TPB, COFs-TP-BD, Fe_3_O_4_@TAPB-TPA, SNW-1	HPLC, GC-MS, HPLC-DAD	[16,27,45,46]
BUs	juice, tea drinks, milk, soybean milk, fruits, vegetable	MSPE, SPE	CMP (CZ-TPB, pyrrole-terephthalde- hyde), COFs (DAAQ-TP, TP-Azo), HAzo-COP (DAAQ-m-trihydroxybenzene)	HPLC	[13,14,47-49]
Fluoroquinolones	pork, chicken, beef	MSPE	Fe_3_O_4_@TPBD@Au-MPS	HPLC-MS/MS	[50]
TCs	grass carp, duck, water	SPE, MSPE	SCU-1@PAN, NiFe_2_O_4_@TAPB-TPA	HPLC	[35,51]
SAs	milk, chicken, pork, beef, chicken, shrimp	SPE, MSPE	SNW-1@PAN, HCCP-DAB, Fe_3_O_4_@TPBD, Fe_3_O_4_@MOF@COF (TFPA-TAPA), Fe_3_O_4_@TPBD@Au-β-CD, Fe_3_O_4_@TAP-4,4'-biphenyl-dicarboxa	LC-MS/MS, HPLC-DAD	[17,33,34,52-56]
EDCs, phyto- hormones	chicken, pork, shrimp, water, beverage, juice, packaged milk, toma- to, grape juice	MSPE, SPE	Fe_3_O_4_@TPBD, COF-TPT, NiFe_2_O_4_@TAPB-TPA, magG@PDA@TbBd, Fe_3_O_4_@TPBD, melamine-paraformaldehyde	HPLC-FLD, GC-MS, GC-MS/MS, GC- FID, HPLC	[31,57-61]
PAHs and their derivates	edible oil, coffee, water, chicken, fish, smoked pork and bacon, slimming tea	MSPE, SPME	Fe_3_O_4_@TPDA, TPBD, Fe_3_O_4_@TPBD, M-TPC-CTFs, COP-QP-TC	HPLC-DAD, HPLC, UPLC-FLD, GC-MS/MS, GC-MS	[22,62-65]
PFASs	milk, water	MSPE, SPME	Fe_3_O_4_@TpPa-F_4_, dioxin-linked COF	UPLC-MS/MS	[24,66]
PCBs	aquatic products	SPME	TFPB-BD	GC-MS/MS	[32]
Tyramine	fermented meats	SPE	MIOP-COF-TpPa	HPLC	[36]
HAAs	fried chicken, roast beef	MSPE	CTC-COF-MCNT	UPLC-MS/MS	[21]
DBPs	drinking water	SPE	TpTt	GC-MS	[67]
Mycotoxin	bean, bean souce	SPE	CCLP-gel	HPLC	[9]
Inorganic ions (N, Br, Cd^2+^, et al)	milk, water	SPE	CTPBD, DhaTab-S, PV-COF	ICP-MS, IC	[17,68,69]
Sudan dyes, FWAs	mushrooms, noodles, wheat flours, sausage, chilli powder	SPE	HL-COPs, MICOPs (Tp-2,6-diaminopyridine)	HPLC-DAD, HPLC	[7,10]

Azo: 4,4-azodianiline; BD: benzidine; CC: cyanuric chloride; CPs: chlorophenols; DA: 2,6-diaminoanthraqsnuinone; DAAQ: 2,6-diaminoanthraquinone; DAB: 3,3'-diaminobenzidine; Da-V: vinyl modified 2,5-dihydroxyterephthalaldehyde; DhaTab-S: surfactant modified COF (Dha Tab-V); Dt: 2,5-dihydroxyterephthalaldehyde; DBPs: disinfection by-products; EDCs: endocrine disrupting chemicals; EDA: electron-donor-electron acceptor interactions; FWAs: fluorescent whitening agents; HCCP: hexachlorocyclotriphosphazene; M-PCTP: magnetic porous covalent triazine-based organic polymer; MICOPs: molecularly imprinted covalent organic polymers; OCPs: organochlorine pesticides; OPPs: organophosphorus pesticides; PAEs: phthalate esters; PAHs: polycyclic aromatic hydrocarbons; PAN: polyacrylonitrile; Pa-NO_2_: 2-nitro-1,4-phenelynediamine; PCBs: polychlorinated biphenyls; PD: p-phenylenediamine; PUs: phenylureas; PV-COF: porphyrin COF with viologen SAs: sulfonamides; SNW: Schiff base networks; SNW-1: COF (melamine-terephthalaldehyde); SCU-1: COF(TMC-PD); TP: 1,3,5-triformylphloroglucinol; TAP: 5,10,15,20-tetrakis(4-aminophenyl)porphyrin; TAPA: tris(4-aminophenyl)amine; TAPB: 1,3,5-tris(4-aminophenyl)benzene; Tab: 1,3,5-tris(4-aminophenyl)benzene; Tb: 1,3,5-tris(4-aminophenyl) benzene; TCs: tetracycline antibiotics; TFPA: tris(4-formylphenyl)amine; TFPB: 1,3,5-tris-(4-formylphenyl)benzene; THB: *m*-trihydroxybenzene; TMC: trimethyl chloride; TPA: triphenylamine; TPA: terephthaldicarboxaldehyde; TPB: 1,3,5-triphenylbenzene; TPC: triptycene; TPT: 2,4,6-triphenoxy-1,3,5-triazine.

## 5 总结和展望

综上所述,多孔质轻的COPs基于其高比表面积而具有强大的吸附性能,在食品安全分析前处理中展示了优异的富集作用。尽管如此,COPs在食品分析样品前处理领域仍有如下方面需进一步研究。1)制备方法:COPs制备方法以高温溶剂热法为主,该方法耗时,反应条件苛刻,试剂毒性高。开发绿色、简单高效的制备方法依旧是COPs的研究基础,高效制备有利于COPs广泛应用。2)功能化COPs设计制备:样品前处理中普通COPs吸附分析物,多为非特异性吸附。在富集目标物的同时,共存化合物也被富集。因而,样品经COPs吸附处理后,虽然降低了基质干扰,但可能增加共吸附化合物对仪器分析的干扰。因此,根据目标物特征,合成或选择含有功能基团的单体,从源头制备功能化COPs;或COPs基质合成后再进行如氨基化、巯基化、离子化等功能化修饰,是将来COPs的一大研究内容。3)材料是否晶型对萃取效果的影响:晶型和无定型材料对萃取效果的影响研究极少,吸附机理仍需深入探讨。4)开拓COPs在分析领域的新应用:COPs一般具有疏水特性,因此,可作为疏水性目标物的吸附剂。吸附目标物后的COPs可直接作为MALDI类质谱分析的进样基质,富集目标物,降低背景信号,同时提高复杂样品的分析通量。或前处理后,与光谱技术联用,提高分析速度和灵敏度。总之,随着材料制备技术的发展和萃取机理的深入研究,COPs在食品安全分析中样品前处理领域将获得越来越广泛的应用。
